# The Working After Cancer Study (WACS): a population-based study of middle-aged workers diagnosed with colorectal cancer and their return to work experiences

**DOI:** 10.1186/1471-2458-11-604

**Published:** 2011-07-29

**Authors:** Louisa G Gordon, Brigid M Lynch, Vanessa L Beesley, Nicholas Graves, Catherine McGrath, Peter O'Rourke, Penelope M Webb

**Affiliations:** 1 Griffith University, Griffith Health Institute, Centre for Applied Health Economics, University Drive, Meadowbrook Q4131, Australia; 2 Queensland Institute of Medical Research, Population Health Department, 300 Herston Rd, Herston, Brisbane Q4006 Australia; 3 Queensland University of Technology, School of Public Health, Victoria Park Rd, Kelvin Grove, Brisbane, Q4006 Australia; 4 Alberta Health Services- Cancer Care, Department of Population Health Research 1331 29th Street NW Calgary T2N 4N2, Canada

## Abstract

**Background:**

The number of middle-aged working individuals being diagnosed with cancer is increasing and so too will disruptions to their employment. The aim of the Working After Cancer Study is to examine the changes to work participation in the 12 months following a diagnosis of primary colorectal cancer. The study will identify barriers to work resumption, describe limitations on workforce participation, and evaluate the influence of these factors on health-related quality of life.

**Methods/Design:**

An observational population-based study has been designed involving 260 adults newly-diagnosed with colorectal cancer between January 2010 and September 2011 and who were in paid employment at the time they were diagnosed. These cancer cases will be compared to a nationally representative comparison group of 520 adults with no history of cancer from the general population. Eligible cases will have a histologically confirmed diagnosis of colorectal cancer and will be identified through the Queensland Cancer Registry. Data on the comparison group will be drawn from the Household, Income and Labour Dynamics in Australia (HILDA) Survey. Data collection for the cancer group will occur at 6 and 12 months after diagnosis, with work questions also asked about the time of diagnosis, while retrospective data on the comparison group will be come from HILDA Waves 2009 and 2010. Using validated instruments administered via telephone and postal surveys, data will be collected on socio-demographic factors, work status and circumstances, and health-related quality of life (HRQoL) for both groups while the cases will have additional data collected on cancer treatment and symptoms, work productivity and cancer-related HRQoL. Primary outcomes include change in work participation at 12 months, time to work re-entry, work limitations and change in HRQoL status.

**Discussion:**

This study will address the reasons for work cessation after cancer, the mechanisms people use to remain working and existing workplace support structures and the implications for individuals, families and workplaces. It may also provide key information for governments on productivity losses.

**Study Registration:**

Australian and New Zealand Clinical Trial Registry No. ACTRN12611000530921

## Background

Although cancer is often seen as a disease afflicting older people, each year in Australia over 40,000 cancers, or 43% of all cancers, are diagnosed in middle-aged people of working ages (45-64 years) [1] and the number of survivors living with cancer is increasing [2]. Consequently, research attention has turned to assessing health-related quality of life (HRQoL) and survivorship issues after a diagnosis of cancer.

One survivorship issue that is not well quantified is work participation and workplace issues after a diagnosis of cancer. Work can define a person's self-worth, identity and social purpose, and it contributes to financial security. A cancer experience that causes major disruption in the work role can become a source of high distress in addition to expensive medical bills, and can adversely affect HRQoL [3,4]. Inability to work may deprive an individual of stimulation, social contacts and independence, while staying in or returning to work after cancer treatment may be important for patients in maintaining a sense of normalcy and control [3,4]. Cancer treatments are improving and current treatments can involve prolonged periods of adjuvant therapy thus previous reports on the extent of disruption to work roles, earnings and other role activities may be outdated. Lengthy treatments, ongoing medical care or the experience of a recurrence may lead to reduced career options and loss of employment. Calls have therefore been made for oncology health workers to better recognise and screen patients for work-related distress [4].

Studies have shown between 30-93% of workers with cancer will return to work [5,6], with most people returning to work within 12 months of taking leave [5,6]. Survivors of head and neck cancer and breast cancer have shown more difficulty returning to work than survivors of other cancer types [5-7]. Although many people appear to resume their employment with minimal interference [8,9], there may also be difficult work re-entry, forced retirement, workplace discrimination and refusal of insurances [10]. Factors that have been linked with delayed return to, or stopping work include: older age [6,11,12]; physically-demanding work [5,6,9,12]; being female [5,6,11,12]; presence of comorbidities [5]; being married [6,11]; fatigue [13]; lower education [6,14]; chemotherapy [11]; blue collar occupations [11]; and upper-body limitations [9]. Amongst those who return to work, work disabilities are more common for those with a physically demanding job, advanced cancer stage and those experiencing treatment side-effects [4]. However, many of these studies are US-based and subject to a system of employment-based health insurance and the added pressure on individuals to keep working to retain access to health care services.

Current research concentrates on breast cancer survivors so the relevance for other cancer populations is unclear. A population-based cohort study of Australian colorectal cancer survivors assessed a subset of working adults for changes in work participation [15]. Twelve months after diagnosis, 33% of men (n = 621) and 40% of women (n = 354) were not working. Radiation therapy among men (OR = 1.90, 95%CI: 1.14- 3.17) and chemotherapy among women (OR = 1.87, 95%CI: 0.98-3.57) were associated with a higher prevalence of work cessation [15]. The risk of ceasing work among women was smaller if they had private health insurance (OR = 0.54, 95%CI: 0.31-0.92). Quality of life scores for persons who stopped working were significantly lower than for persons who continued working, after adjusting for additional explanatory factors [15]. However, this study was limited because there was no non-cancer comparison group and therefore was unable to determine the proportion of cases who would have ceased work irrespective of cancer.

This paper presents the protocol of a population-based observational study to examine the work experiences in adults with colorectal cancer. We aim to describe changes in work participation at two points in time within a 12-month period, identify the key predictors influencing work participation and time to work re-entry, quantify the extent of physical and cognitive limitations at work and the role of work on HRQoL. The results of the study will provide valuable information for individuals facing cancer, health professionals, supportive care services and government about the reasons for work cessation, the mechanisms people use to remain working and existing workplace support structures.

## Methods

### Study design

A longitudinal population-based study has been designed to enrol middle-aged (45-64 years) men and women newly-diagnosed with colorectal cancer who were working at the time they were diagnosed. These participants will be matched by gender and 5-year age group to a nationally representative sample of men and women from the general population. Participants in both groups will be followed over 12-months and have data collected on socio-demographic factors, work-related factors, and HRQoL while the cancer sample will have additional data collected on cancer treatment and symptoms, work productivity and cancer-related HRQoL.

### Aims and hypotheses

The key aims of the study are to:

1. Describe *transitions *in employment participation following a primary diagnosis of colorectal cancer within a 12-month period compared to individuals without cancer;

2. Identify the key factors influencing *work participation *during or after cancer treatment compared to individuals without cancer;

3. Identify the key factors influencing *time to work re-entry *after cancer treatment among individuals taking work leave for their cancer treatment;

4. Investigate the influence of changes in employment participation over a 12-month period on *HRQoL *at time 2 among individuals with cancer compared to those without cancer; and

5. Quantify the extent of *physical and cognitive limitations *at work (work disability) in individuals following a primary diagnosis of colorectal cancer.

We hypothesise that:

1. The proportion of middle-aged working adults with a primary diagnosis of colorectal cancer who substantially alter their work hours (i.e. by ≥20%) or stop working by 12 months after diagnosis will be more than 15% higher than among middle-aged working adults in the general community;

2. The key barriers to work participation or timely work re-entry after cancer will include regional or metastatic cancer at diagnosis, age, fatigue at six months, adjuvant therapy, living with an employed partner, limited work autonomy and low income; and,

3. Study participants who substantially reduce their work hours or leave the work force against their choice, will have clinically lower HRQoL scores at 12 months compared to those who remain employed at 12 months.

### Study participants

Eligible cases will be Queensland residents, aged 45-64 years, with a histologically confirmed diagnosis (notified to the Queensland Cancer Registry (QCR)) of primary colorectal cancer between January 2010 and September 2011, and in paid employment at the time of their cancer diagnosis. The QCR has universal coverage of persons diagnosed with cancer residing in Queensland. Study exclusions include: cognitive impairment; not speaking English; having no telephone; and previous or concurrent cancers (except non-melanoma skin cancer). A general population comparison group will be randomly selected from a secondary data source, the Household, Income and Labour Dynamics in Australia (HILDA) Survey dataset, using retrospective data in Waves 9 and 10 (2009 and 2010 data). HILDA is an Australian household panel survey which began in 2001 and collects information about economic and subjective well-being (including HRQoL), labour market and family dynamics. Households and individuals are followed up annually through interviews and self-administered questionnaires. Each new survey wave of HILDA, is developed and pilot-tested over a 9-month period using a sample of urban and rural households prior to data collection.

Ethics approval for the study was obtained from the Human Ethics Research Committees of Queensland Institute of Medical Research, Griffith University and the Queensland Health Research Ethics and Governance Unit.

### Sample size

Based on cancer incidence [16] and labour figures [17] it is anticipated that at least 522 working persons with colorectal cancer will be eligible annually. Sample size calculations were based on the number required to detect a 15% difference in work participation between workers with and without cancer assuming a 5% significance level and 80% power. Based on our past experience of recruiting colorectal cancer patients through the QCR and allowing for 85% doctors consent, 70% participant consent, 90% baseline interview completions and 75% retention at 12 months post-diagnosis, we will require initial contact with 258 patients with colorectal cancer to retain 125 patients at 12 months. The wave-on-wave attrition rates among continuing HILDA respondents have been consistently low, approximately 5% each year [18]. Data for 2,590 employed persons enrolled in HILDA aged 45-64 years Australia-wide will be available. HILDA data will be randomly matched 2:1 to patients with colorectal cancer, by gender and 5-year age category. Thus the total sample size to be selected for the study will be approximately 780.

### Recruitment

As mandated by QCR protocols, patients' names and the names of their doctors will be accessed via the QCR and initial letters sent to each patient's doctor requesting permission for the patient to be approached, followed by reminder letters and telephone calls if necessary. All patients for whom doctor's consent is obtained will be mailed a study invitation letter, an information sheet and consent form. Three weeks after the initial patient letter, non-responders will be telephoned to gauge their interest in the study and screened for their eligibility.

### Data collection sources

Data will be collected during this project from four sources (Table 1):

**Table 1:** Questionnaire item domains at each time point by cancer cases and general-population comparison group

	Cancer Group		Comparison Group
**Method**	**Telephone interview**	**Mailed questionnaire**	**Face-to-face interview**

Time 1^a^	1. Socio-demographics^b^ 2. Cancer treatments 3. Symptoms (MSAS^b^) 4. Work questions^a^ 5. Work limitations (WLQ)	6. Quality of life (SF-36)^b^ 7. Health behaviours & conditions^b^ 8. Cancer-specific quality of life (FACT-C) 9. Financial strain^b^	Question groups 1, 4, 6, 7, 8, 9 from HILDA Wave 9, 2009 data

Time 2	Repeat question groups 2-8		Question groups 1, 4, 6, 7, 8 from HILDA Wave 10, 2010 data

^a ^Time 1 for the Cancer Group occurs six-months after diagnosis however retrospective work questions are asked about their time of diagnosis (baseline) and about their current time (six months after diagnosis) therefore a common one year period will be compared across the cancer and comparison groups for key work questions.

^b^These questions are the same as those in the comparison group to enable direct comparison between groups.

Abbrevs: FACT-C = Functional Assessment in Cancer Therapy plus Colorectal Cancer module, HILDA = Household, Income and Labour Dynamics in Australia, MSAS = Memorial Symptom Assessment Scale, SF-36 = Medical Outcomes Study Short Form 36, WLQ = Work Limitations Questionnaire.

#### 1) Pathology reports held within the QCR (cancer group)

Clinical data will be collected including tumour site, histopathological tumour type, degree of differentiation/grade, degree of metastasis and stage of disease (e.g, TNM or Dukes staging).

#### 2) Telephone interviews (cancer group)

Participants with cancer will take part in structured telephone interviews at 6 and 12 months after diagnosis. At the 6-month data collection time point we will also ask participants to recall work-related information at the time they were diagnosed with cancer (baseline). Telephone interviews will be undertaken in order to maximise response rates, reach a wide geographical region and minimise participant burden, the latter being important in a working population that may require out-of-hours contact. Interviews will be conducted by trained and experienced interviewers. To minimise respondent burden, interviews will be kept to approximately 30 minutes each. A flexible interview schedule will be adopted and tailored to suit the schedules of working participants.

#### 3) Self-administered mailed questionnaire (cancer group)

To supplement the data collected from the telephone interview, and to address items of a more sensitive nature (e.g., financial strain, health behaviours), additional postal surveys will be sent to participants with cancer immediately after the telephone interview. The surveys will take approximately 20 minutes to complete.

#### 4) General-population (comparison group) survey data

Secondary data from HILDA survey waves 9 and 10 (2009 and 2010 data) will be used to assess the comparison group. These data are collected by face-to-face interviews.

### Specific questionnaire items and measures

*Generic baseline socio-demographic information*for the cancer and comparison groups will include items on age, gender, education level, country of birth, culture, marital status, household income, health and other insurances, postcode of residence, number and age of children.

*Participants' work situations, generic HRQoL, health behaviours and conditions*will be collected at both time-points (Table 1) with identical items for the cancer and comparison groups. Work situation items include a compilation of validated tools from Australian government surveys (eg Australian Bureau of Statistics (ABS) Labour Force Survey, ABS 1999 Survey of Living Standards) and ask about: employment status, usual and preferred weekly hours, reasons for working part time, occupation, occupation change, industry, trade union membership, paid sick leave, paid holiday leave, expected resignation or dismissal, supervisory responsibilities, employer type, workplace size, job satisfaction, if the respondent has a disability, type of disability, disability commenced in the last year, impact of disability on work probability, financial strain (e.g., access to emergency funds, bills paid late), intended age of retirement and intentions of stopping work during next three years. Generic HRQoL will be evaluated using the widely-used and validated Medical Outcomes Survey Short Form (SF-36). Physical activity estimates will be obtained via the International Physical Activity Questionnaire (short-form) [19]. Other standard health behaviours and conditions (smoking, alcohol consumption, height, weight and chronic illnesses) will also be asked.

*Cancer treatments, symptoms, cancer-specific HRQoL and work limitation items*will be collected at both time-points for participants in the cancer group (Table 1). The Memorial Symptom Assessment Scale (MSAS) will be used as our measure of prevalence and severity of common cancer treatment and disease symptoms [20]. This instrument has proven validity and reliability among a sample comprising persons with prostate, breast, colon and ovarian cancers [20]. Cancer-specific HRQoL will be assessed using the Functional Assessment of Cancer Therapy general questionnaire (FACT-G) plus the colorectal cancer module; (FACT-C). The FACT-G (Version 4) is a 36-item questionnaire with four subscales: physical; social; emotional; and functional well-being. The FACT-C has demonstrated validity and reliability, is brief and sensitive to changes in functional status [21]. In addition, Queensland general-population norms are available to use for comparisons [22]. The 25-item Work Limitations Questionnaire (WLQ) will be used to assess potential difficulties experienced at work [23]. The WLQ covers four dimensions; time management, physical demands of work, mental-interpersonal demands (cognitive job tasks, on-the-job social interactions) and output demands (diminished work quantity and quality). The WLQ has strong psychometric properties and has tested in patients with various chronic diseases [23].

### Analyses

All analyses will include gender as a factor and will test for interactions of gender with other factors in the model. Gender differences are important, as not only do men and women have different work participation rates, work hours and occupation types in the general population [17], they respond and adjust differently to illnesses, have different health behaviours and other risk factors for cancer [24]. Where no effect modification is observed, pooled results will be presented. Socio-demographic characteristics will be compared at baseline to identify any statistically significant differences between the cancer and comparison groups. Descriptive analyses will be conducted to chart employment transitions over the year since diagnosis among the cancer group and over 2009-2010 year period among the comparison group. Although the first time point for the cancer group is six months after diagnosis, participants are asked to recall work circumstances (occupation type, hours worked, industry etc) at the time of diagnosis (retrospectively) and at time 1 (six months after diagnosis). Separate multivariable logistic regression modelling will identify explanatory variables that are significantly associated with work participation in the cancer and comparison groups. For these models, adjusted odds ratios with 95% confidence intervals and p-values will be computed. Cox proportional hazards models will assess time to work resumption in the participants with cancer and significant correlates of timely work re-entry. Mixed-effects modelling for the cancer group will be used to examine change in HRQoL scores (time 1 - time 2) by participants who reduce or leave in that time versus those who do not. We will also compare HRQoL scores between the cancer and comparison groups at time 2. Data on work limitations measured in the cancer group will be scored across the four domains and mixed-effects modelling undertaken to assess changes in scores between time 1 and time 2. Reasons for study withdrawal in the cancer sample will be closely monitored. Descriptive analyses of baseline variables for the 'completers' and 'non-completers' will identify any possible bias and the source and, if necessary, subsequent analyses will be weighted accordingly. In the comparison group, only responders at both HILDA Waves 9 and 10 will be included.

We expect the relationship between work participation and HRQoL to be complex and therefore, to complement the analyses above, statistical modelling will be undertaken to allow for health status to be an endogenous component of work participation. We will address this using the estimation of two-stage simultaneous probit least squares [25]. Unlike previous analyses examining the relationship between health status and work participation [25,26] in patients with chronic illness, we will have comprehensive clinical data to describe disease status in addition to self-reported health status. STATA SE (Version 11.0) will be used for all analyses. Written reports of the study findings will adhere to the STROBE (Strengthening the Reporting of Observational studies in Epidemiology) statement for quality purposes.

## Discussion

Despite the considerable number of Australians of working ages being diagnosed with cancer each year, surprisingly little is known about the impact of cancer on the workforce. With an ageing population, the Australian Government has recently implemented policies to encourage people to remain working longer and has increased access to personal superannuation funds and social security benefits (e.g. pension bonus schemes). The challenges presented to 'baby boomers' (those born during 1946-1961) differ from those facing previous generations because, as they approach normal retirement age, they may face caring responsibilities for both elderly parents and young grandchildren [27]. Female baby boomers may be disadvantaged financially due to working in occupations that were traditionally low-paid and female-dominated (i.e., nursing, secretarial, retail, teaching) and before equal pay legislation (pre-1960s) and compulsory superannuation laws. They may also have taken longer periods off work to raise families, compared to subsequent generations, and so collectively may have little retirement savings and financial security [27]. Middle-aged men with cancer may be at the height of their careers, and their sense of self may be strongly attached to their work roles. Therefore, cancer may negatively affect a person's employment and financial situation and fuel the anxiety and distress created from facing a life-threatening illness.

Our research will extend the current literature and improve our understanding in this field. The translation of current knowledge into supportive care practice is limited because of the use of retrospective or cross-sectional designs and/or, patient groups from a single clinical setting, a dominance of breast cancer among the patient samples and, importantly, the inability to isolate the effects of ageing, retirement choice and labour force changes due to the lack of a non-cancer control group. Our study will overcome these limitations by recruiting a population-based sample and using validated instruments, including a non-cancer comparison group and utilising both a prospective and retrospective design. We have chosen colorectal cancer because this cancer type is the most common to both men and women, and we will have a homogenous disease group from which to compare men and women.

The study will address an important issue in cancer survivorship in greater detail than previously reported and, in doing so, will provide further insights into living with cancer and the potential implications for individuals, families and workplaces. It will also advance methods in economic evaluations of health care where a better understanding of productivity losses is gaining importance. Further, this project will identify specific issues and barriers faced by cancer survivors who want to remain employed, and provide practical information to supportive cancer care providers able to directly translate this information into developing resources for newly diagnosed cancer patients.

## Competing interests

The authors declare that they have no competing interests.

## Authors' contributions

LG conceived of the study, and participated in its design and coordination and drafted the manuscript. BL, CM and VB participated in the design of the study and will assist in the statistical analyses and written reports of the findings. PO, NG and PW have contributed to the study design and continue to oversee the study performance to ensure the successful completion of the study. All authors have read and approved the final manuscript.

## Pre-publication history

The pre-publication history for this paper can be accessed here:

http://www.biomedcentral.com/1471-2458/11/604/prepub
